# Does the route of immunoglobin replacement therapy impact quality of life and satisfaction in patients with primary immunodeficiency? Insights from the French cohort “Visages”

**DOI:** 10.1186/s13023-016-0452-9

**Published:** 2016-06-22

**Authors:** B. Bienvenu, G. Cozon, C. Hoarau, M. Pasquet, P. Cherin, P. Clerson, E. Hachulla, J. C. Crave, J. C. Delain, R. Jaussaud

**Affiliations:** Internal Medicine Department, University Hospital Centre of Caen, avenue de la Côte de Nacre, BP 95182, 14033 Caen cedex 9, France; Edouard Herriot Hospital, Clinical Immunology, 5 place d’Arsonval, 69437 Lyon cedex 03, France; Renal Transplantation & Clinical immunology Department, University Hospital Centre of Tours, 2 bd Tonnellé, 37044 Tours cedex, France; Pediatric Hematology and Oncology Department, University Hospital Centre of Toulouse, 330 avenue de la Grande Bretagne, 31059 Toulouse cedex 9, France; Internal Medicine Department, Saint Antoine Hospital, 184 rue du Faubourg Saint Antoine, 75571 Paris cedex 12, France; Soladis Clinical Studies, 84 boulevard du Général Leclerc, 59100 Roubaix, France; Internal medecine Department, CHRU Lille – Hôpital Claude Huriez, 2 avenue Oscar Lambret, 59037 Lille Cedex, France; Octapharma France, 62bis avenue André Morizet, 92100 Boulogne-Billancourt, France; Internal Medicine and Infectious Diseases Department, University Hospital Centre of Reims, avenue du Gal Koenig, 51092 Reims cedex, France

**Keywords:** Primary immunodeficiency, Immunotherapy, Replacement, Immunoglobulins, Quality of life, Satisfaction, Preference, Cohort study

## Abstract

**Background:**

IgG replacement therapy (IgRT) in primary immunodeficiencies (PID) is a lifelong treatment which may be administered intravenously (IVIg) or subcutaneously (SCIg), at hospital or at home. The objective of the VISAGE study was to investigate if route and/or place for IgRT impact patients’ satisfaction regarding IgRT and quality of life (QoL) in real-life conditions.

**Methods:**

The study enrolled PID patients at least 15 years old receiving IgRT for at least 3 months. Satisfaction and QoL were evaluated at enrollment and over a 12-month follow-up period by Life Quality Index (LQI) which measures 3 dimensions of satisfaction: treatment interference, therapy related problems and therapy settings (factors I, II and III) and SF-36 v2 questionnaire.

**Results:**

The study included 116 PID patients (mean age 42 ± 18 years, 44 % males, 58 % with scholar or professional occupation) receiving IgRT for a mean of 8.5 ± 8.4 years. At enrollment they were receiving either home-based SCIg (51 %), hospital-based IVIg (40 %) or home-based IVIg (9 %). Patients exhibited a high degree of satisfaction regarding IgRT whatever the route and place for administration. LQI factor I was higher for home-based SCIg (86 ± 2) than for hospital-based IVIg (81 ± 3) and home-based IVIg (73 ± 5; *p* = 0.02 versus home-based SCIg); no difference was found for LQI factor II; LQI factor III was higher for home-based SCIg (92 ± 2) than for hospital-based IVIg (87 ± 5) and hospital-based IVIg (82 ± 3; *p* = 0.005 versus home-based SCIg). By contrast, every dimension of QoL was impaired. Over the follow-up period, 10 patients switched from hospital-based IVIg to home-based SCIg and improved LQI factor I (*p* = 0.004) and factor III (*p* = 0.02), while no change was noticed in LQI factors II and QoL. Meanwhile, no change in satisfaction or QoL was found in patients with stable route of IgRT. When asked on their preferred place of treatment all but one patient with home-based treatment would choose to be treated at home and 29 % of patients treated at hospital would prefer home-based IgRT.

**Conclusion:**

PID patients expressed a high degree of satisfaction regarding IgRT, contrasting with impaired QoL. In real-life conditions awareness of patient’s expectations regarding the route or place of IgRT may be associated with further improvement of satisfaction.

## Background

Primary immunodeficiency diseases (PIDs) encompass a large group of rare heterogeneous genetic diseases with various degrees of impairment of innate or adaptive immune systems. Most of them like agammaglobulinemia, X-linked agammaglobulinemia, common variable immune deficiency (CVID), severe combined immunodeficiency (SCID), and immunoglobulin subclass deficiency associated with recurrent infections, are characterized by a low level of circulating immunoglobulins (Ig). Ig deficiencies expose the patient to longer, more frequent, and more severe infections, affecting mainly lungs and bowel [1]. PIDs have a negative impact on personal and professional activities, social relationships, fatigue and anxiety thus impairing quality of life [2–9]. The French National Reference Center for Primary Immune Deficiencies (CEREDIH) has recently issued recommendations about the prevention of infections [10] promoting the role of Ig replacement therapy (IgRT) and protection from infection. IgRT increases the level of circulating IgG [11], prevents infections, prolongs survival and enhances quality of life [3, 12–17]. The residual circulating IgG target concentration is 8 g/L in practice [10]. IgRT is usually a life-long treatment except for patients with SCID in whom IgRT is indicated during the period before curative treatment (allogenic hematopoietic stem cell transplant or gene therapy). IgRT is commonly administered by intravenous (IVIg) or subcutaneous route (SCIg). IVIg are infused at 3-to-4-week intervals over 2-4 h, most often at hospital, even if IVIg treatment is possible at home with the intervention of a third person. SCIg are administered usually once or twice weekly over 1–2 h, mostly at home; the use of compact programmable pumps allows the patient to keep moving and attending his/her usual daily activities. Generally, SCIg require more frequent injections of a smaller volume but with lower impact on the patient’s activity and no requirement of a venous bed of good quality. IVIg require monthly injections which last longer and have a greater impact on patient’s activities. IVIg and SCIg share similar efficacy in preventing infections [18–21] but IVIg are responsible for supraphysiological peaks in serum IgG concentrations immediately after the infusion, followed by rapid fall in the next few days and progressive decrease over 3 to 4 weeks [21–23]. On the other hand, SCIg allow more stable serum IgG levels between injections [17, 22, 24]. Local reactions are more frequent with SCIg whereas general systemic reactions are more often observed with IVIg [14, 18, 22, 25–27], a point that could be explained by supraphysiological Ig peaks [17]. Both routes have comparable efficacy in the prevention of serious bacterial infections [28]. In addition, SCIg are more cost-effective through a reduction of lost work days [29]. Home-based treatment seems to be associated with better quality of life when compared to hospital-based IgRT, whatever the administration route is [30]. Patients’ preference is, however, not univocal. Some patients prefer to receive injections at hospital because of the ease of organization and the quality of care and follow-up; while others prefer home-based treatment due to the lower impact on daily activities. These dimensions are encompassed in the general concept of satisfaction regarding the treatment [31].

## Methods

### Objectives

The objective of the VISAGES study was to describe, over a 12-month follow-up period and in real-life conditions, patients’ satisfaction regarding IgRT and QoL in regard to modalities of IgRT (IVIg or SCIg, hospital-based or home-based treatment).

### Ethics

The non-interventional nature of the research protocol was confirmed by the French Ethics Committee *“Comité de Protection des Personnes - Ile-de-France V”*. The study protocol and related documents (CRF, informed consent form) gained approval from the French Medical Research Data Processing Advisory Committee *(“Comité Consultatif sur le Traitement de l’Information en matière de recherche dans le domaine de la Santé”, CCTIRS)*. The French Information Technology and Privacy Commission *(“Commission Nationale de l’Informatique et des Libertés”, CNIL)* approved the research protocol and related data collection.

### Methods

The VISAGES study was a prospective, non-interventional cohort study conducted in France in PID patients, who were receiving IgRT for at least 3 months at the time of enrollment and who were planning to pursue IgRT for at least 12 further months. Patients who were participating in a clinical trial could not be enrolled. Patients were recruited by hospital centers highly experienced in the management of PIDs and were followed up under real-life conditions for 12 months after enrollment. Given its observational nature, the study did not modify the usual medical care of the patients. The type, dose, and route of IgRT were entirely left up to the physician’s discretion.

Collected data included demographics, body mass index, lifestyle, occupation and past and concomitant diseases. The type, number and severity of infectious events within the 12 months preceding enrollment were reported at enrollment and prospectively collected over the follow-up period. Severe infections were defined as meningitis, pneumonia, sepsis, osteitis, or visceral abscess. History of IgRT was collected. IgG serum concentration was reported when monitored. Patients’ satisfaction was assessed by the Life Quality Index (LQI) [32], a self-administered questionnaire which comprises three independent factors named treatment interference (factor I), therapy related problems (factor II), and therapy settings (factor III). Quality of life was assessed at enrollment and at each visit by the self-administered SF-36 v2 scale in patients over 15 years and the self-administered CHQ-PF50 questionnaire in younger patients. For SF-36 v2, using norm-based scoring, each health domain scale and summary of physical and psychosocial health measures, from 0 (worse health) to 100 (better health) were scored to have the same mean (50) and standard deviation (10) as in the general US population. Compliance was prospectively evaluated by questions related to potential difficulty to be supplied with immunoglobulins and the occurrence of delayed or missed injections between visits. The patient was instructed to fill in a diary after each immunoglobulin injection providing details on the route of administration and on local and general reactions. At enrollment, patients were asked about their preferred place for IgRT administration if they could choose it.

We report results in patients at least 15 years old, which is the lower age for the use of the SF-36 scale.

### Statistics

Sample size calculation was based on the estimation of the mean of each factor of LQI. Assuming a common standard deviation of 25 for each factor [32], a sample size of 139 patients was required to estimate each mean with a two-sided 95 % confidence interval of 4. Quality of life and patients’ satisfaction regarding IgRT was evaluated using a mixed model with the place (hospital or home) and the route for administration (IVIg or SCIg), and place by route interaction as fixed factors and center as random factor. Contrasts between groups were estimated along with their 95 % confidence interval (CI). The type 1 error risk has not been adjusted. Analyses have been performed independently for each of the 3 LQI factors, the physical component, the mental component and every dimension of the SF-36 v2 questionnaire. Changes were calculated as last documented value (endpoint) minus baseline. Changes were compared between patients who switched from IVIg to SCIg during the follow-up period and those who did not change the modalities of IgRT. Due to their small number, no analysis could be performed for patients who switched from SCIg to IVIg. Within-group changes were tested with signed test for paired data. Between-groups comparisons used analyses of variance with adjustment for baseline value. Same analyses were conducted between patients who changed from hospital-based to home-based IgRT over the follow-up period and those whose place for IgRT remained unchanged. The annual incidence rate of infections was estimated by a negative binomial model with Pearson’s scale using the logarithm of the follow-up duration (in years) as an offset term. The statistical analysis was conducted with the SAS 9.3. software (SAS Institute Cary, NC, USA).

## Results

### Population

At 35 hospital centers 116 patients who were at least 15 years of age were enrolled. Patient characteristics are detailed in Tables 1 and 2. Patients were 15 to 84 years old, 44.0 % were males. 19.0 % were living alone and 57.4 % had a professional occupation or frequented school. PID was diagnosed more than 10 years before the inclusion (median 6.6 years) and IgRT was started 8.5 ± 8.4 years prior to inclusion (median 5.6 years). Patients were suffering from agammaglobulinemia (*n* = 1), X-linked agammaglobulinemia (*n* = 5), hypogammaglobulinemia (*n* = 9), CVID (*n* = 76), severe combined immunodeficiency (*n* = 4), IgG subclass deficiency (*n* = 12) or of other types of PID (*n* = 9). 81 patients were suffering from at least one concomitant disease. Most frequent or relevant comorbidities were anemia in 8 patients, fibromyalgia in 6 patients, psoriasis in 6 patients, asthma in 4 patients, thyroiditis and ankylosing spondyloarthritis both in 3 patients, and interstitial lung disease in 2. One patient was deaf. Six patients had experienced at least one severe infection within the previous 12 months. Among them, two were receiving IgRT for less than 1 year. Since IgRT initiation, 49 patients had switched at least once from IVIg to SCIg and 14 patients had switched at least once from SCIg to IVIg. IVIg to SCIg switches were driven by a combination of patient’s request (*n* = 19), poor venous access (*n* = 5), desire to preserve venous access (*n* = 7),patient’s activity (*n* = 20), or other reasons (*n* = 22). SCIg to IVIg switches were due to a combination of patient’s request (*n* = 8), poor local tolerance (*n* = 2), desire to decrease the frequency of infusions (*n* = 3), or other reasons (*n* = 6). At the time of enrollment, 50.6 % of patients were receiving SCIg at home, 40.0 % were receiving IVIg at hospital and 9.4 % were receiving IVIg at home (Table 3). The current route was ongoing for 8.6 ± 9.3 years for IVIg and 3.4 ± 2.1 years for SCIg. The median IVIg dose was 571 mg/kg/month whereas the median SCIg dose was 428 mg/kg/month. The mean trough IgG level was 8.8 ± 2.2 g/L at last determination before enrollment.

**Table 1:** Population

	Number		
Age (years)	116	41.8 ± 17.5	[15-84; 39.5]
BMI (kg/m^2^)	116	23.4 ± 4.7	[15.2-40.2; 22.3]
Males	51	44.0 %	
Females	65	56.0 %	
Living alone	22	19.0 %	
School of professional occupation	66/115	57.4 %	

Frequencies are presented as n/N with N being the number of documented data

**Table 2:** History of PID

	*N* = 116		
Age at PID diagnosis (years)		31.6 ± 20.3	[0–79.6; 32.4]
Time from PID diagnosis (years)		10.2 ± 9.9	[0.4–50.6; 6.6]
Type of PID			
• Agammaglobulinemia	1	0.9 %	
• X-linked agammaglobulinemia	5	4.3 %	
• Hypogammaglobulinemia	9	7.8 %	
• Common variable immune deficiency	76	65.5 %	
• Severe combined immuno-deficiency	4	3.5 %	
• IgG subclass deficiency with recurrent infections	12	10.3 %	
• Other PID	9	7.8 %	
At least one severe infection within the previous 12 months	6	5.2 %

**Table 3:** History of IgRT

	*N* = 116		
Age at start of IgRT (years)	115^a^	33.34 ± 20.17	[0–79.7; 33.6]
Duration of IgRT (years)	115^a^	8.5 ± 8.4	[0.2–38.5; 5.6]
History of at least one switch from IVIg to SCIg	49	42.2 %	
History of at least one switch from SCIg to IVIg	14	12.1 %	
Route of IgRT at enrollment			
• IVIg at hospital	46	39.7 %	
• IVIg at home	13	11.2 %	
• SCIg at home	57	49.1 %)	
Switch from IVIg to SCIg after enrollment	11	9.5 %	
Switch from SCIg to IVIg after enrollment	2	1.7 %	
Switch from hospital-based to home-based IgRT	12	10.3 %	
Switch from home-based to hospital-based IgRT	2	1.7 %	

^a^missing data for one patient

### Ig replacement therapy

**Fig. 1:**
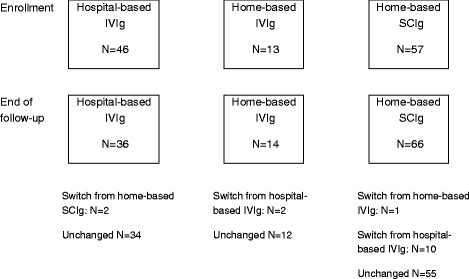
Change in route and/or place of IgRT administration over the study

Patients were followed up for 12.6 ± 3.6 months after enrollment. Compliance was excellent with only 11 patients (7.8 %) having missed at least one infusion during the study period. During follow-up, the modalities of IgRT remained unchanged for 101 (87.1 %) patients and were changed for 15 patients. Ten patients switched from hospital-based IVIg to home-based SCIg, 2 patients with IVIg switched from hospital to home treatment, one patient changed home-based IVIg to home-based SCIg and 2 patients switched from SCIg at home to IVIg at hospital (Fig. 1). Therefore, change in route was often associated with change in place of administration. Changes from hospital-based to home-based IgRT was mainly motivated by patient’s request, opportunity of collaboration with a service provider and/or a private nurse, and patient’s good understanding of home-based treatment advantages and disadvantages. Changes from home-based to hospital-based IgRT were less frequent (*N* = 4). Two were related to poor local tolerance of SCIg. 82.4 % of patients respected the scheduled injections during the 12-month follow-up period.

### Satisfaction regarding IgRT

At enrollment, patients exhibited high levels of satisfaction regarding IgRT (Table 4). Satisfaction regarding treatment interference (LQI Factor I) was higher for home-based SCIg than for home-based IVIg (*p* = 0.02) but not higher than for hospital-based IVIg (*p* = 0.19). In patients with IVIg the place for administration had no impact on LQI Factor I (*p* = 0.12). No impact of route and place for administration was found on satisfaction regarding therapy related problems (LQI Factor II). Satisfaction regarding therapy setting was maximal for home-based SCIg (*p* = 0.005 when compared to hospital-based IVIg). In patients with home-based IgRT, no difference was found between IVIg and SCIg (*p* = 0.37). The presence of comorbidity had no impact on the satisfaction level.

**Table 4:** Satisfaction regarding IgRT at enrollment

	LQI Factor I	LQI Factor II	LQI Factor III
Hospital-based IVIg	81.2 ± 2.5	85.8 ± 2.3	82.3 ± 2.5
Home-based IVIg	72.8 ± 4.7	79.5 ± 4.1	86.9 ± 4.5
Home-based SCIg	85.6 ± 2.3	85.6 ± 1.9	91.5 ± 2.2
Hospital-based IVIg vs home based IVIg	[-2.3 to 19.0] *P* = 0.12	[-2.8 to 15.5] *P* = 0.17	[14.8 to 5.5 *P* = 0.37
Hospital-based IVIg vs home based SCIg	[-11.1 to 2.2] *P* = 0.19	[-5.5 to 6.0] *P* = 0.93	[-15.5 to -2.8] *P* = 0.005
Home-based IVIg vs home based SCIg	[-23.3 to -2.3] *P* = 0.02	[-15.1 to 2.9] *P* = 0.18	[-14.6 to 5.4] *P* = 0.37

Results are expressed as mean ± standard error. Contrasts are derived from a mixed model with route, place and route by place interaction as fixed factor and center as random factor. Contrasts are provided as two-sided 95 % confidence interval and *p* value

Ten patients switched from IVIg to SCIg during follow-up with subsequent improvement in LQI Factor I (11.0 ± 13.7, *p* = 0.04, signed test). No change was noticed for LQI Factor II. Improvement in LQI Factor III did not reach significance (*p* = 0.12). Meanwhile no change was found in patients with unchanged route of administration. Comparing these two groups of patients, the difference in LQI Factor I and LQI Factor III were significant (*p* = 0.004 and *p* = 0.02). Since almost all patients who switched from IVIg to SCIg concomitantly changed from hospital-based to home-based treatment, similar results were found when comparing change in LQI between patients who replaced hospital-based by home-based treatment (data not shown).

### Quality of life

At enrollment all dimensions of quality of life, assessed by the SF-36 v2 questionnaire, were impaired (Table 5). No difference was found between places or routes for IgRT. Quality of life remained unchanged in the 10 patients who switched from IVIg to SCIg during follow-up and no difference was found at the end of the follow-up period between patients who changed and those who did not change IgRT modalities. Patients with concomitant disease had significantly lower scores in some dimensions of quality of life: physical functioning, role physical, bodily pain, and social functioning. Generally, there was no impact of comorbidities on the mental component but alterations of the physical component.

**Table 5:** Quality of life (SF36 v2) at inclusion regarding IgRT modalities

	Hospital-based IVIg *N* = 46	Home-based IVIg *N* = 13	Home-based SCIg *N* = 57	*p*
Physical functioning	47.61 ± 9.78	48.79 ± 12.74	49.11 ± 9.97	0.76
Role physical	44.66 ± 10.04	48.54 ± 8.91	45.24 ± 11.39	0.56
Bodily pain	47.13 ± 10.63	49.63 ± 11.62	46.75 ± 13.81	0.78
General health	37.39 ± 10.35	34.34 ± 10.04	37.70 ± 10.38	0.61
Vitality	44.11 ± 11.03	48.69 ± 10.02	45.38 ± 10.19	0.43
Social functioning	43.15 ± 11.25	40.98 ± 11.03	43.52 ± 13.51	0.83
Role emotional	41.28 ± 14.29	40.33 ± 11.53	44.29 ± 13.89	0.47
Mental health	42.19 ± 12.56	42.07 ± 13.78	40.58 ± 12.36	0.81
Physical component score	46.25 ± 9.39	49.41 ± 9.04	46.94 ± 10.48	0.64
Mental component score	40.93 ± 13.70	39.51 ± 13.01	41.42 ± 12.98	0.91

Mixed model with route and place and interaction of route by place for administration as fixed factors and study center as random factor

### Infections

Ten patients experienced a total of 16 severe infections, mostly pulmonary, during the follow-up period resulting in an incidence rate of 0.19 per patient-year [95 % CI 0.08–0.46] without significant difference between IVIg and SCIg. The IgG level was monitored at least once during follow-up in 100 patients for a total of 203 determinations. At only three occasions, serum IgG concentrations of less than 5 g/L were reported.

### Patient’s preference

At enrollment, 11 patients (29 % of those who expressed a preference) receiving hospital-based IVIg declared to prefer being treated at home. Among them 6 switched from hospital-based IVIg to home-based SCIg during follow-up. One patient (8.3 %) treated at enrollment by home-based IVIg preferred being treated at hospital. He switched to home-based SCIg. No patients with SCIg treatment at home expressed their preference for hospital-based IgRT. Two of them however switched to hospital-based IVIg during follow-up.

## Discussion

This observational cohort study involving PID patients receiving IgRT for a long time showed that patients had high levels of satisfaction regarding IgRT with better results for treatment interference and therapy setting (LQI factors I and III) in patients receiving home-based SCIg. In patients receiving home-based treatment, SCIg was associated with a higher level of satisfaction regarding treatment interference when compared to IVIg; in patients receiving IVIg, the place for administration did not affect satisfaction with regard to treatment interference. No impact of route and place for administration was found on satisfaction regarding therapy related problems (LQI Factor II). The satisfaction regarding therapy setting was higher in patients receiving home-based treatment. Contrasting with high levels of satisfaction, quality of life was impaired in every dimension of the SF-36 questionnaire. Over the 12-month follow-up, 10 patients switched from hospital-based IVIg to home-based SCIg and improved LQI factor I (*p* = 0.004) and factor III (*p* = 0.02) while no change was noticed in LQI factors II and QoL. Meanwhile no change in satisfaction or QoL was found in patients with a stable route of IgRT. When asked about their preferred place of treatment, all but one patient with home-based IgRT would choose to be treated at home and 29 % of patients treated at hospital would prefer home-based IgRT. The annual incidence rate of severe infections was 0.19 per patient-year [95 % CI 0.08–0.46], without difference between IVIg and SCIg.

### Modalities of IgRT

At the time of enrollment 50.6 % of patients were receiving SCIg at home, 40.0 % were receiving IVIg at hospital and 9.4 % were receiving IVIg at home. Similar numbers have been recently reported in a study of 216 PID patients and 84 caregivers recruited in 21 countries [31]: hospital-based IVIg (47 %), home-based IVIg (7 %), hospital-based SCIg (3 %) and home-based SCIg (43 %). The importance of SCIg in France has already been highlighted. In 2006 the IRIS study group found that 58 % of French PID patients were receiving SCIg [33]. Conversely, the analysis of the European ESID database in 2008 showed that 76 % of patients were receiving IVIg [34, 35].

### Patients’ satisfaction

Patient’s satisfaction is a key factor of compliance. Satisfaction is related to experience and expectations. The higher the correlation between them, the better the patient’s satisfaction. At the same efficacy level, the choice between two treatments or two administration routes may depend on patients’ satisfaction [36]. SCIg injections are more frequent than IVIg and sometimes involve multiple sites, two points that could impair patients’ satisfaction [37]; on the other hand, monthly travels to hospital could also be a source of dissatisfaction for the patient. The life quality index (LQI) aims at evaluating a patient’s satisfaction exploring 3 dimensions: treatment interference, therapy-related problems and therapy settings. LQI has been developed for patients with IgRT [32]. Evaluating satisfaction of 58 PID patients receiving lifelong IgRT, Nicolay et al., [15] found values for factors I, II and III of 90.3 ± 12.8, 80.9 ± 20.3 and 96.1 ± 7.8. Results showed slightly lower satisfaction in our population specially when considering that 67 % of Nicolay’s patients were receiving hospital-based IVIg and that 13 % were receiving home-based IVIg, two groups of patients who exhibited lower satisfaction levels for factors I and III in our population. Our results are more in line with a study reported by Gardulf et al. [38] in 21 children and 85 adults receiving home-based SCIg with mean values of 86.4, 78.4 and 93.2 for factors I, II, and III, respectively after 10 months of treatment. Using another tool for measuring satisfaction by a questionnaire sent to the patients and caregivers affiliated to the International Patient Organisation for Primary Immunodeficiencies (IPOPI), Espanol et al. [31] reported that most patients (76 %) were satisfied with their treatment, with higher levels of satisfaction in patients receiving SCIg than in those treated with IVIg. Treatment interference with daily life was lower with SCIg, and patients highlighted the ability to self-administer, being able to fit the treatment into their schedule and the reduced duration of administration. Higher satisfaction levels have already been attributed to better therapy convenience and greater independence [3].

Ten patients switched from IVIg to SCIg during the follow-up. Change of route was most often combined with change of place. In the Nicolay’s study [15], 39 patients switched from hospital-based IVIg to home-based SCIg with a significant improvement of factor I and factor III. In the Gardulf study [38], 77 patients switched from hospital-based SCIg to home-based SCIg with a significant improvement of LQI factors I, II and III. In our study, switching from IVIg to SCIg was followed by a significant improvement in factors I and III. As in the Nicolay study no change was reported in factor II. Interestingly, satisfaction levels remained stable in patients who did not change the modalities of IgRT during follow-up. Since changes in route of administration were mostly concomitant to changes in place for administration, we were not able to discriminate the role of change in route or place on satisfaction improvement.

### Quality of life

IgRT has demonstrated improvement of quality of life in PID patients mainly by reducing the frequency of infections and the fear of further infections [12, 39]. Despite the low incidence of infectious events in our population with long-standing IgRT, all dimensions of quality of life were impaired. This has already been reported. Patients with CVID were found to score lower on the General Health scale in comparison to other patients with chronic diseases and to have higher limitations due to their physical health [7]. Children with PID had significantly lower emotional and social functioning compared to children with juvenile inflammatory arthritis [6]. Asking PID patients and/or their caregivers about satisfaction regarding IgRT and quality of life (SF-12 v2), Espanol et al. [31] reported an impairment in quality of life with scores ranging from 37.9 (general health) to 46.5 (mental health). The summary physical component was 40.7 and the mental component was 46.5. Presence of comorbidities impaired physical components of quality of life. Fairly poor quality of life contrasted with high levels of satisfaction. The absence of correlation between quality of life and satisfaction has already been reported [15]. These results suggest that the SF-36 measures different underlying concepts than the LQI and more generally than satisfaction cannot be confounded with quality of life. Switch from IVIg to SCIg improved satisfaction regarding IgRT but did not modify QoL measured by the SF-36 v2. Similar results have already been reported. In a study of 40 patients switching from IVIg to SCIg no difference was seen in SF-36 for patients over 14 years whereas the mean LQI significantly improved [40].

### Patients’ preference

Around 70 % of patients with hospital-based IgRT confirmed their preference for hospital-based treatment whereas almost all patients with home-based treatment would choose to be treated at home if given the choice. These results suggest that IgRT takes advantages of various places and routes for administration and that patients should have access to the most appropriate immunoglobulin therapy according to their disease and personal conditions. Espanol et al. [31] conducted a conjoint analysis of patients’ preference based on five attributes of the treatment (autonomy regarding administration, frequency, place, duration of administration and number of needle sticks). They reported that patients and caregivers generally preferred self-administration over administration by a health care professional and administration at home rather than at hospital. These results were however driven by patients receiving SCIg at home, and patients treated with IVIg preferred an administration by a health professional. Studying 30 patients having switched from IVIg to SCIg, Hoffman et al. reported that 92 % of adults stated a preference for SCIg and 83 % preferred home therapy over therapy in clinic setting [41]. The physician should certainly be aware of the patient’s preference regarding the modalities of IgRT to improve patient’s satisfaction. Moreover, patients should be informed and involved in the choice of the most appropriate and best individualized treatment. In the study conducted by Espanol et al. [31] only 13 % of patients have been the main decision-makers in the choice of IgRT modalities.

### Limitations

This study has some limitations. Due to its observational nature, visits were not prescheduled. Infectious events have been collected retrospectively by the physician at each visit. Residual IgG levels were not monitored on a regular basis. Switches from IVIg to SCIg or from hospital-based to home-based treatment were decided in real-life conditions and were not randomized. Therefore, the group of patients whose modalities of IgRT remained unchanged cannot be considered as a control group. Completion of patient’s diaries was not exhaustive and could have been more likely completed by patients who were more prone to complain from adverse reactions. Patients were recruited by specialized hospital centers and could therefore not be representative of the whole population of PID patients receiving IgRT in France. Nevertheless the inclusion/exclusion criteria were not expected to select a specific population.

## Conclusion

PID patients with long-standing IgRT expressed a high degree of satisfaction regarding the replacement therapy. Conversely, all dimensions of quality of life were impaired. Satisfaction regarding treatment interference and therapy settings was higher for SCIg at home. A significant proportion of patients however expressed their preference of hospital-based treatment. These results suggest that the physician should be aware of the patient’s preference and personal conditions when choosing the modalities of IgRT. Sharing the decision with the patient would improve the patient’s satisfaction regarding IgRT.
